# Emerging Nanomedicine Strategies for Chronic Disease Management Based on Chitosan

**DOI:** 10.3390/ijms27031387

**Published:** 2026-01-30

**Authors:** Yaride Pérez-Pacheco, Deepak Parajuli, Ricard García-Valls

**Affiliations:** Department of Chemical Engineering, Universitat Rovira i Virgili, Campus Sescelades, Av. Països Catalans 26, 43007 Tarragona, Spain; yaride.perez@urv.cat (Y.P.-P.); deepak.parajuli@urv.cat (D.P.)

**Keywords:** chitosan nanocarriers, chronic disease, encapsulation, personalized medicine, targeted drug delivery

## Abstract

Chronic diseases such as cancer, cardiovascular disorders, neurodegenerative conditions, chronic respiratory diseases, autoimmune disorders, chronic kidney disease, persistent infectious diseases, diabetes, and ocular inflammation remain leading causes of morbidity and mortality worldwide. Their complex pathophysiologies and the limitations of conventional therapies underscore the urgent need for advanced drug delivery platforms that enhance therapeutic efficacy while minimizing off-target effects and systemic toxicity leading to adverse reactions. Nanomedicine has emerged as a transformative approach, with chitosan-based nanocarriers offering advantages due to their biocompatibility, biodegradability, mucoadhesive properties, and ability to be physic-chemically modified. These nanocarriers improve solubility, stability, bioavailability, and the therapeutic index of drugs, while enabling controlled release, targeted delivery, and immune modulation. This review highlights recent advances in chitosan-based nanomedicine for the management of chronic disease. We discuss methods of synthesis such as ionic gelation and electrospray, functionalization approaches, and immunomodulatory roles that expand therapeutic potential. The evidence emphasizes that chitosan nanocarriers are a versatile, safe, and effective platform which can be used to improve clinical results, reduce adverse effects, and advance the science of personalized medicine.

## 1. Overview

Cancer, cardiovascular diseases, neurodegenerative disorders, chronic respiratory diseases, rheumatoid arthritis and other autoimmune diseases, chronic kidney disease, infectious diseases with a prolonged course, diabetes, and ocular inflammation are chronic diseases that represent some of the most persistent global health challenges. These diseases contribute to morbidity, mortality, and reduced quality of life worldwide [1]. This review highlights the advantages of chitosan for encapsulating and delivering the active agents, safely and efficiently, for different disease treatments, which will likely result in further pharmacokinetic and clinical investigations. The information reviewed here shows the potential of chitosan-based nanocarriers as a safe and effective platform for enhancing different therapies and minimizing adverse effects while maximizing treatment benefits, due to the drug loading, controlled release, and enhanced targeting that together alleviate disease symptoms and ultimately improve patient outcomes.

Cancer is characterized by uncontrolled cell proliferation, evasion of apoptosis, and the capacity to invade tissues and metastasize. Its complex pathology requires multimodal therapeutic approaches, including surgery, chemotherapy, radiation, and targeted therapies that often carry severe side effects and face the challenge of drug resistance [2,3,4]. Cardiovascular diseases, such as coronary artery disease, heart failure, and stroke, are determined by risk factors like hypertension, atherosclerosis, and diabetes, requiring chronic management to prevent severe events [5]. Neurodegenerative diseases, including multiple sclerosis [6], Alzheimer’s disease [7] and Parkinson’s disease, involve progressive neuronal loss leading to cognitive and motor dysfunction, with a limited number of disease-modifying treatments currently available [8]. Chronic respiratory diseases like chronic obstructive pulmonary disease [9] and asthma [10] cause persistent airway inflammation and airflow limitation, resulting in reduced lung function and frequent worsening, which can negatively affect daily life. Autoimmune diseases such as rheumatoid arthritis trigger chronic inflammation and joint destruction through abnormal immune responses, often requiring immunosuppressive therapies that carry risks of infection [11,12]. Chronic kidney disease is characterized by gradual loss of renal function, often leads to end-stage renal disease requiring dialysis or transplantation, and is closely linked with cardiovascular complications [13]. Infectious diseases with chronic progressions, including HIV/AIDS and tuberculosis, remain major public health concerns due to their persistence, transmission potential, and the challenges in adherence to associated long-term treatments [14]. Diabetes mellitus is a metabolic disorder defined by reduced glucose metabolism, and it causes extensive systemic complications such as cardiovascular disease, neuropathy, nephropathy, and retinopathy. Effective management demands permanent glycemic control through medications, lifestyle changes, and monitoring, yet poor patient adherence and suboptimal drug bioavailability complicate treatment results [15,16]. Ocular inflammations like uveitis, conjunctivitis, and keratitis can result in irreversible tissue damage and vision loss if left untreated. Treatment effectiveness is often limited by anatomical and physiological barriers including resultant injuries, corneal permeability, and poor drug retention on the ocular surface, presenting significant challenges for topical therapies [17].

These chronic diseases highlight the urgent need for improved therapeutic strategies and targeted drug delivery systems as the era of nanomedicine approaches. These drug delivery systems must improve their efficacy, reduce systemic toxicity, enhance patient adherence, and overcome the biological barriers inherent to these conditions [18].

### 1.1. Nanocarriers Enhance Drug Delivery

**Figure 1:**
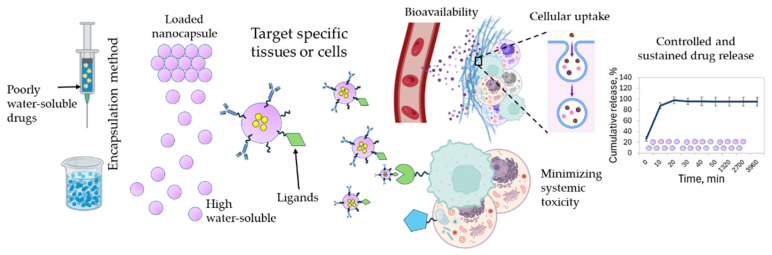
Mechanisms and advantages of nanocapsule-based drug delivery.

Nanomedicine is a new approach in modern therapeutics, specifically for the management of chronic diseases that require precise and effective drug delivery. Nanocarriers offer several advantages over traditional drug formulations, mainly due to their nanoscale size and physicochemical properties [18]. Figure 1 shows the mechanisms and advantages of nanocapsule-based drug delivery. These nanocarriers can improve the solubility and stability of poorly water-soluble drugs, enhance bioavailability, increase the ability to cross biological barriers, increase the potential for codelivery and enable controlled and sustained drug release, thus preserving therapeutic drug levels over prolonged periods [19]. Nanocarriers can be engineered to target specific tissues or cells, reducing off-target effects and minimizing systemic toxicity [18,20]. Such targeted delivery is helpful for potent bioactive compounds that have limited therapeutic opportunities [21]. Nanocarriers can protect sensitive drugs from early degradation in the biological environment. Their surface can be modified with ligands or polymers such as chitosan to enhance mucoadhesion, cellular uptake [22], and immunoactivation without toxic effects [23]. Therefore, nanomedicine is a strategy which can be used to overcome many pharmacokinetic and pharmacodynamic limitations of conventional therapies, by improving patient outcomes and thereby opening the way for advancements in personalized medicine.

Clinical acceptance and the commercialization of nanoencapsulation technologies for drug delivery remain limited due to several critical scientific, regulatory, economic, and educational barriers. So far, liposomes [24] and some polymeric micelles [25] have reached the commercial market. The complexity of human biology presents significant challenges for scientific and technical investigation; even well-engineered, targeted nanocarriers that demonstrate efficacy in vivo or in animal models may be quickly cleared by the immune system, accumulate in non-target organs, or fail to reach the target tissue in therapeutic concentrations. This is specifically problematic in oncology, where tumor heterogeneity, variability within and between tumors in the same patient, complicates the development of generalized targeting strategies [2,26].

### 1.2. Introduction to Chitosan as a Versatile Biopolymer for Nanocarriers

Chitosan-based encapsulation systems are considered to be viable drug delivery systems for the treatment of chronic disease. These systems improve the oral, intravenous bioavailability and sustained release of therapeutic agents, addressing challenges in drug solubility, stability, and absorption; they are considered to be biologically superior, compared to other natural polymers, as shown in Table 1. Moreover, chitosan-based nanocarriers have shown potential in gene therapy applications, and the application also represents a non-toxic delivery system.

Chitosan is a natural polysaccharide obtained from the deacetylation of chitin found in the exoskeleton of crustaceans [43]. It has gained significant attention as a nanocarrier material for drug delivery systems due to its tunable physicochemical properties, including molecular weight (MW), degree of deacetylation (DD) (details shown in Table 2), surface charge and the pH-responsiveness evident in the protonation of chitosan amines under acidic microenvironments (tumor, inflamed tissue, and endosomes) that triggers swelling or drug release, but can also induce premature protonation in gastric or lysosomal compartments, which can lead to burst release and off-target exposure [44,45]. Encapsulation within chitosan-based nanostructures has been shown to enhance the solubility, stability, and bioavailability of therapeutic agents [46]. Chemical modification strategies such as carboxymethylation or thiolation improve chitosan solubility at physiological pH and contribute to enhanced pharmacokinetic performance, including prolonged circulation time and improved oral absorption, as has been demonstrated for peptides and small-molecule drugs [46,47,48]. Furthermore, chitosan-based nanoparticles protect encapsulated cargos from chemical and enzymatic degradation, enabling sustained and controlled drug release profiles that reduce burst effects and improve the therapeutic index [47,49,50,51]. Its ability to encapsulate a wide range of bioactive compounds, protect them from degradation, and facilitate sustained and controlled release has established chitosan as a support material in the development of advanced nanomedicine platforms targeting chronic diseases such as cancer [48,51,52,53], cardiovascular diseases [54,55,56], neurodegenerative disorders [57,58], chronic respiratory diseases [59,60], rheumatoid arthritis and other autoimmune diseases [61,62,63,64,65,66,67,68,69,70,71,72,73,74,75], chronic disease [49,76,77,78,79,80,81,82,83], diabetes [50,56,84,85,86,87,88,89,90,91], ocular inflammatory diseases [92,93,94,95,96,97] and infectious diseases with a prolonged course [60].

This review paper aims to critically evaluate the potential of chitosan-based nanocarriers as versatile drug delivery platforms for chronic diseases, highlighting their capacity for sustained and controlled release. By analyzing diverse therapeutic contexts, including cancer and cardiovascular, neurodegenerative, respiratory, autoimmune, metabolic, ocular, and chronic infectious diseases, this review identifies the ways in which chitosan encapsulation can overcome pharmacokinetic and biological barriers, improve bioavailability, reduce systemic toxicity, and enhance treatment efficacy. The goal is to provide an integrated understanding of chitosan’s role in nanomedicine, emphasizing mechanistic insights and translational potential relevant to personalized and targeted chronic disease management.

## 2. Fabrication of Chitosan-Based Nanocarriers

Chitosan’s chemical and biological properties make it suitable for drug delivery applications [87,102]. Its biocompatibility ensures that it is well-tolerated by the body, minimizing the risk of adverse immune reactions or toxicity. Chitosan is biodegradable, allowing it to be naturally broken down into non-toxic components by enzymes such as lysozyme [103], which facilitates safe clearance from the body after delivering its therapeutic load. Chitosan’s mucoadhesive ability is due to its positive charge under acidic conditions [104]; this positive charge enables strong electrostatic interactions with the negatively charged mucosal surfaces found in tissues like the gastrointestinal tract, nasal cavity and ocular mucosa, leading to prolonged residence time and enhanced drug absorption [104]. The positive charge of chitosan also promotes the formation of nanoparticles through ionic interactions with negatively charged molecules like sodium tripolyphosphate, enabling the encapsulation and controlled release of drugs [105,106].

Several techniques are commonly employed to synthesize or fabricate chitosan nanoparticles in mild conditions, with different advantages. Ionic gelation is one of the most widely used methods, relying on the electrostatic interaction between the positively charged amino groups of chitosan and negatively charged crosslinking agents such as sodium tripolyphosphate [45,107,108]. This mild and simple technique allows for the formation of stable nanoparticles under aqueous conditions without the need for harsh chemicals or high temperatures.

**Figure 2:**
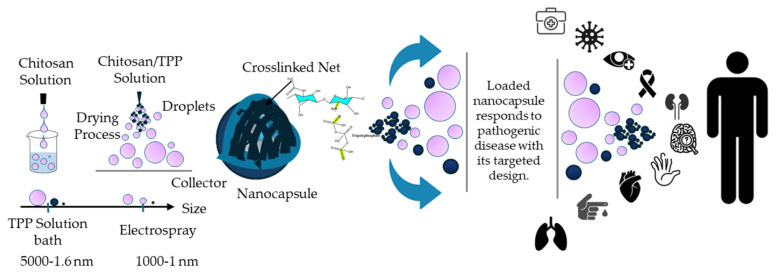
Chitosan-based nanocarriers for targeted drug delivery across major chronic diseases, including cancer and cardiovascular, neurodegenerative, respiratory, autoimmune, renal, infectious, metabolic, and ocular inflammatory disorders.

Electrospray is an innovative technique that uses an electric field to atomize a polymeric solution into fine droplets, which then rapidly solidify into nanoparticles [109,110,111]. This fabrication method allows for precise control over particle size and morphology and is especially useful for producing uniform nanoparticles with great encapsulation efficiency and sustained release [112,113]. Other techniques, such as emulsion crosslinking [114] and spray drying, are also used, depending on the formulation requirements, but ionic gelation and electrospray remain ideal due to their simplicity and their ability to preserve the biological activity of encapsulated compounds. Figure 2 shows the mechanism of chitosan-based nanocapsules used for targeted disease therapy.

### Immunomodulatory Functionalization of Nanoparticles

The primary challenge for chitosan nanoparticles in drug delivery is to improve their circulation time in the bloodstream and ability to accumulate at target sites. Biomimetic design strategies are employed to imitate biological structures and functions, including adsorption techniques and the design of protein coronas; also, membrane-mimetic coatings are used to reduce opsonization and macrophage uptake. In general, nanoparticles are often recognized as foreign by the immune system. However, biomimetic nanoparticles may integrate cell membrane components, targeting ligands and polymeric molecules within the integrated system [27,28].

**Figure 3:**
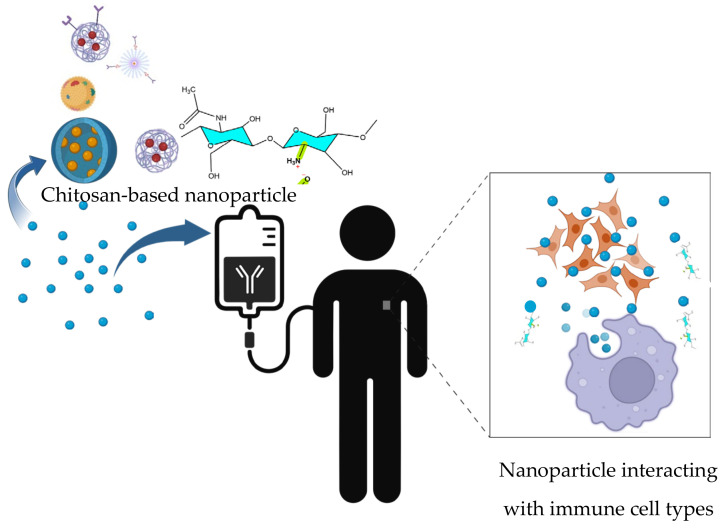
Surface functionalization and immunomodulatory coating strategies for nanoparticles.

In this context, nanoparticles must exhibit active immunomodulatory capacity rather than obtaining only passive foreign body responses [29,30]. Their physicochemical properties, such as size, surface charge, and composition, determine whether they stimulate or suppress immune responses [31]. Nanoparticles can interact with macrophages, neutrophils, and the complement system, leading either to inflammation and cytokine release or, on the contrary, to reduced activation and immune tolerance [32]. Also, their interaction with biomolecules may affect the adaptive response by promoting or inhibiting antibody production and T cell activation. Through these mechanisms, nanoparticles can act as immunostimulatory agents (improving vaccine efficacy or anticancer immunity) [33,34,35] or as immunosuppressive carriers (reducing unwanted inflammation or autoimmune reactions) [36,37]. Figure 3 shows surface functionalization and immunomodulatory strategies using nanoparticles.

The spontaneous formation of protein coronas on nanoparticles has become an approach used to control their biological behavior and therapeutic properties. Rather than simply acting as passive surface layers, these coronas can experience structural reorganizations that dynamically influence cellular signalling, allowing for targeted immune modulation. For instance, nanoparticles self-assembled from aloin were shown to specifically interact with myotrophin through multivalent hydrogen bonds, inducing a conformational shift that reorganized the myotrophin interactome in microglial cells. This structural reconfiguration assisted the release of peroxiredoxin 6, reduced mitochondrial oxidative stress, and effectively inhibited the cGAS–STING inflammatory pathway. These observations emphasize the potential of combining natural compound-based self-assembly with protein corona engineering to create immunomodulatory nanoparticle coatings. Connecting such dynamic conformational behavior supports the controlled polarization of microglia from a pro-inflammatory (M1) to a neuroprotective (M2) phenotype for nanomedicine-driven immune therapies in neurodegenerative disorders [38].

Chitosan’s biocompatibility, biodegradability, and tunable charge enable the fabrication of nanocarriers with controlled size, stability, and drug release profiles. Mild fabrication techniques preserve bioactivity while allowing scalable and versatile nanoparticle design. Additionally, advanced surface functionalization and immunomodulatory strategies further position chitosan-based nanocarriers as flexible platforms for targeted and immune-responsive therapies in chronic diseases.

## 3. Scientometric Analysis of Chitosan-Based Nanomedicine for Chronic Disease Management

For this review, a systematic and stringent literature search was performed to obtain high-quality and up-to-date information on emerging chitosan-based nanomedicine strategies for chronic disease management. Particular emphasis was placed on studies prioritizing in vivo investigations and, when in vivo data were unavailable, well-validated in vivo studies relevant to translational development were used. In parallel, to provide a comprehensive scientometric overview of the research landscape, a single, carefully optimized search query was applied across all fields in the Scopus database (Elsevier). This approach was selected to ensure data consistency, reproducibility, and compatibility; analysis was performed using the VOSviewer software, while maintaining the total number of saved records below the software’s analytical threshold of 20,000 publications. Thus, the final search equation used for bibliometric mapping and trend analysis was defined, as described in the following.

Initially, a scientometric analysis was conducted using the Scopus database (Elsevier) to evaluate the evolution and research trends in chitosan-based nanomedicine for chronic disease management. Many comprehensive search equations were applied, and the results saved included original research articles, review papers, conference abstracts, and patents related to emerging nanomedicine strategies for chronic disease management based on chitosan.

**Figure 4:**
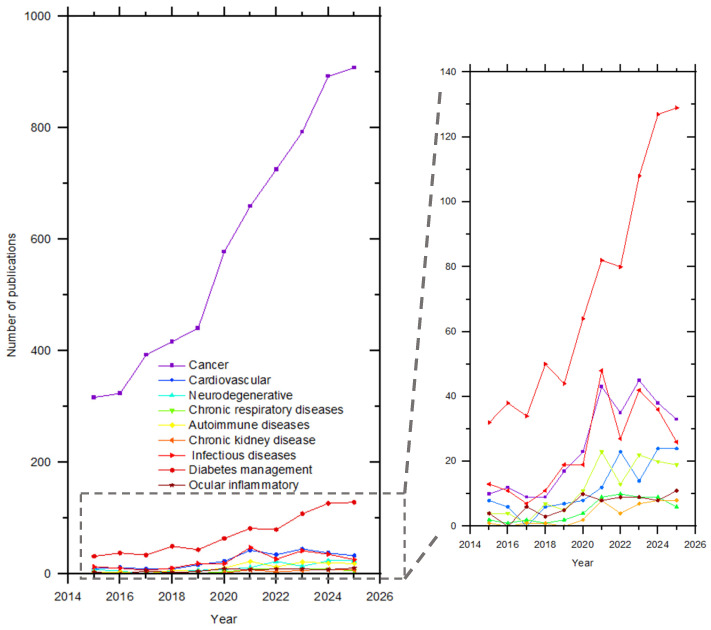
Temporal evolution of scientific publications on chitosan-based nanomedicine for chronic disease management, from 2015 to 2025. Data was retrieved from the Scopus (Elsevier) database, with a consultation date of 15 January 2026. The search was performed across all fields using the following query: TITLE-ABS-KEY (chitosan AND “drug delivery” AND (cancer OR tumor OR oncology OR cardiovascular OR neurodegenerative OR respiratory OR pulmonary OR autoimmune OR “rheumatoid arthritis” OR “chronic kidney disease” OR renal OR infectious OR antimicrobial OR diabetes OR insulin OR ocular OR ophthalmic)). Total number of publications: 12,030.

The optimized search shown in Figure 4 was performed using the following query in all fields: TITLE-ABS-KEY (chitosan AND “drug delivery” AND (cancer OR tumor OR oncology OR cardiovascular OR neurodegenerative OR respiratory OR pulmonary OR autoimmune OR “rheumatoid arthritis” OR “chronic kidney disease” OR renal OR infectious OR antimicrobial OR diabetes OR insulin OR ocular OR ophthalmic)). A total of 12,030 documents were identified. The analysis reveals a marked increase in the number of publications over the last decade, reflecting the growing scientific interest in improving conventional pharmacological formulations through advanced drug delivery strategies. This trend is closely associated with the need to enhance the therapeutic efficacy, biocompatibility, bioavailability, and targeted delivery of drugs used in the long-term treatment of chronic diseases.

As shown in Figure 4, a pronounced growth in publications is observed from 2020 onwards, coinciding with the post-COVID-19 period. This increase can be partially attributed to the accelerated development of nanotechnology-based drug delivery systems, particularly those employed in vaccine formulations, which significantly boosted interest in polymeric carriers. Although cancer-related applications dominate the publication landscape, a consistent upward trend is also evident for other chronic conditions, including cardiovascular diseases, neurodegenerative disorders, chronic respiratory diseases, rheumatoid arthritis and other autoimmune diseases, chronic kidney disease, infectious diseases with a prolonged course, diabetes, and ocular inflammation. Overall, this review highlights the central role of chitosan as a versatile, biocompatible, and functional polymer in emerging nanomedicine strategies, particularly for the design of advanced drug delivery systems aimed at chronic disease management.

**Figure 5:**
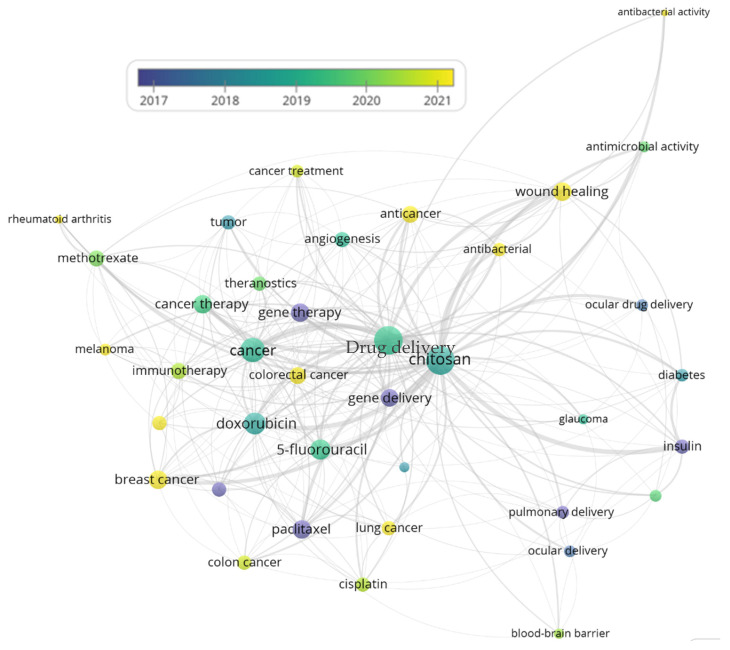
VOSviewer overlay visualization map illustrating keyword co-occurrence and temporal evolution of chitosan-based drug delivery systems for chronic disease management.

Figure 5 presents an overlay visualization map, generated using VOSviewer, based on 12,030 documents saved from the Scopus database using the search query mentioned before. The map illustrates the co-occurrence network of leading keywords, where each node represents a frequently occurring term, node size reflects the relative frequency of occurrence, and links indicate co-occurrence relationships between keywords within the same publications. The spatial proximity of nodes reflects the strength of their conceptual association.

In Figure 5, chitosan and drug delivery emerge as the central connected nodes, confirming their fundamental roles in linking multiple therapeutic areas. Surrounding these central nodes, several disease-specific clusters are observed, namely, cancer-related terms (e.g., breast cancer, lung cancer, colon cancer, chemotherapy, and targeted therapy), as well as keywords associated with diabetes, pulmonary delivery, ocular delivery, antimicrobial activity, and rheumatoid arthritis, highlighting the broad applicability of chitosan-based nanocarriers across chronic disease domains. The overlay color gradient from purple/blue to yellow represents the average publication years of the documents in which each keyword appears. Earlier research topics are shown in cooler colors, whereas more recent research topics appear in warmer colors. Particularly, recent keywords in yellow tones are associated with targeted drug delivery, antimicrobial activity, wound healing, and ocular drug delivery, indicating a shift toward more specialized, application-driven nanomedicine strategies in recent years.

On the whole, the overlay visualization shows a temporal evolution of the research focus, transitioning from fundamental chitosan-based delivery systems toward advanced, disease-specific, and targeted nanomedicine applications, particularly after 2020. This trend aligns with the growing demand for biocompatible, multifunctional nanocarriers for chronic disease management and highlights the increasing relevance of chitosan in emerging nanomedicine platforms, as this review intends to communicate.

The scientometric findings support the finding of this review that chitosan-based nanomedicine is transitioning from a predominantly exploratory research field to a more application-driven and translational discipline. The evidence positions chitosan as a key material for next-generation drug delivery systems aimed at improving therapeutic outcomes in chronic disease management, while emphasizing the necessity for coordinated efforts to accelerate clinical translation.

## 4. Applications

### 4.1. Applications for Cancer Therapy

Advances in nanotechnology may upgrade cancer therapy from drug delivery and gene silencing to environmental responsiveness and targeted cytotoxicity. Nanoparticles may overcome limitations of conventional treatments like systemic toxicity, poor tumor selectivity, and rapid clearance of drug carriers by the immune system [29]. Within this context, chitosan enables the rational design of nanocarriers with enhanced tumor accumulation, controlled drug release, and reduced off-target effects.

**Figure 6:**
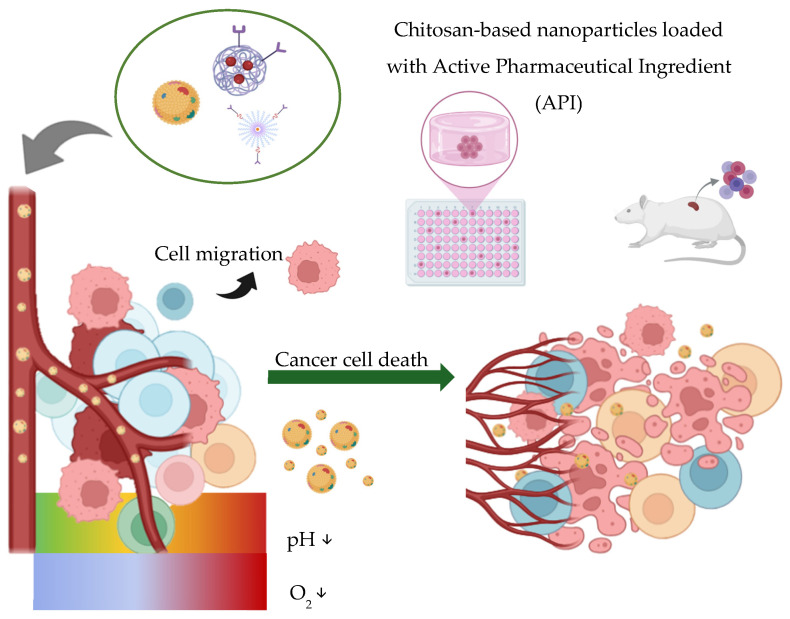
Chitosan-based nanoparticles as used in applications in cancer therapy.

This section reviews representative studies employing chitosan either as a nanocarrier matrix or as a functional surface component used to improve therapeutic efficacy, specificity, and safety in cancer treatment. Chitosan-based nanoparticles can interact with tumor tissues through multiple mechanisms, including electrostatic cell membrane interactions, receptor-mediated uptake, microenvironment-responsive release, and intracellular trafficking modulation. Rather than functioning solely as passive carriers, these systems actively participate in overcoming biological barriers associated with tumor progression and treatment resistance. Figure 6 represents the ways in which chitosan is used in various applications in cancer therapy.

As summarized in Table 3, chitosan-based nanocarriers support a spectrum of therapeutic strategies, ranging from conventional drug delivery and pH-responsive systems to multifunctional platforms and gene- or RNA-based therapies. Targeted carriers such as FA-PMAN/CS nanoparticles [53] demonstrate how surface functionalization improves pharmacokinetics and tumor localization through receptor-mediated uptake and immune evasion. In parallel, pH-responsive formulations use the acidic tumor microenvironment to enable selective drug activation, thereby enhancing cytotoxicity while limiting systemic exposure [115,116,117]. More complex architectures integrate multiple therapeutic modalities, such as reactive oxygen species generation, metabolic interference, photothermal effects, and gene regulation, to address tumor heterogeneity and resistance mechanisms [48,52]. Significantly, chitosan-based systems also enable advanced gene and RNA delivery approaches, including non-invasive pulmonary administration and blood–brain barrier penetration for glioblastoma treatment [102,118].

These studies show the possibilities of chitosan for nanotechnology-based applications in advancing cancer therapy; by integrating biomimetic camouflage, environmental responsiveness, gene modulation, and natural carrier systems, researchers are developing increasingly precise and effective treatments. Although these advances have been made, challenges such as large-scale manufacturing, regulatory approval, and long-term biosafety must be addressed to enable clinical translation. However, the future of cancer therapy is likely to be shaped by these innovative nanomedicine platforms, opening the way for personalized and targeted oncological care.

### 4.2. Applications in Cardiovascular Diseases

**Figure 7:**
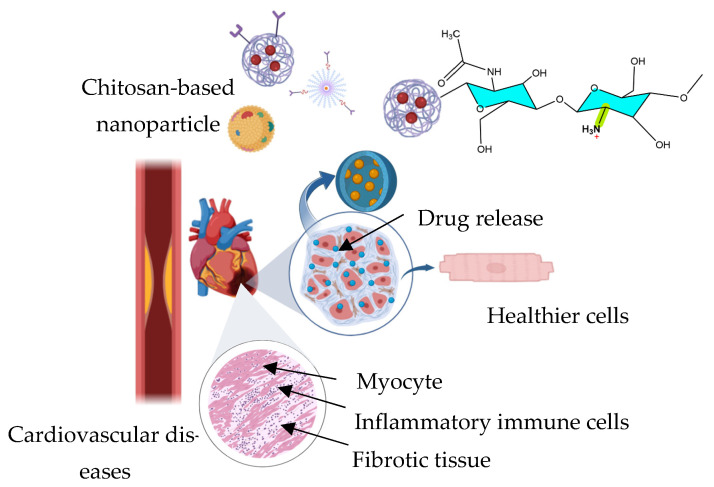
Chitosan-based nanoparticles used in applications in cardiovascular diseases.

Nanocarriers based on chitosan also enhance the delivery of therapeutics in various cardiovascular diseases like hypertension, atherosclerosis, or heart failure with improved targeting and reduced side effects. Chitosan-based nanoparticles, hydrogels, and microparticles have been engineered to deliver antihypertensive agents, anticoagulants, statins, and gene therapies, allowing for controlled and sustained release at targeted sites such as atherosclerotic plaques or an infarcted myocardium [56]. Moreover, chitosan, being pH-responsive, can be functionalized to improve its targeting capability and responsiveness to stimuli such as oxidative stress, key factors in cardiovascular pathophysiology [119]. Figure 7 shows chitosan-based nanoparticles used for applications in cardiovascular diseases.

One approach is encapsulating agents that mitigate myocardial damage and cardiac fibrosis into chitosan-based encapsulation systems addressing cardiovascular complications associated with sepsis. Nanoparticles composed of astragalus polysaccharides (APS) encapsulated within a chitosan/tripolyphosphate (CS/TPP) matrix have demonstrated cardioprotective effects in septic conditions. These APS-CS/TPP nanoparticles reduced bacterial load, serum C-reactive protein, white blood cell counts, and cardiac troponin I levels, key markers of systemic inflammation and cardiac injury. Also, they mitigated myocardial histopathological damage and preserved cardiac function by modulating inflammatory responses. Mechanistically, these nanoparticles suppressed the TLR4/NF-κB signaling pathway, a central mediator in the overproduction of inflammatory cytokines such as TNF-α, IL-1β, MCP-1, and IL-6, which are implicated in septic cardiomyopathy [119].

Chitosan-based encapsulation also addresses cardiac fibrosis, a pathological feature common in various cardiovascular diseases, including ischemic heart failure, diabetic cardiomyopathy, and age-related cardiac decline. Chitosan/TPP nanoparticles have been loaded with ginsenoside Rb3 (G-Rb3), a bioactive compound with known effects on energy metabolism but limited oral bioavailability. The resultant formulation demonstrated a therapeutic effect by modulating myocardial energy metabolism through activation of the peroxisome proliferator-activated receptor alpha pathway. This mechanism improved fatty acid oxidation, glycolysis, and mitochondrial oxidative phosphorylation, ultimately reducing myocardial fibrosis [56].

In the context of myocardial infarction, injectable chitosan hydrogels, especially those integrated with conductive polymers like polypyrrole-grafted gelatin, have demonstrated support in myocardial repair by enhancing cell viability, mimicking the mechanical and electrical properties of cardiac tissue, reducing inflammation, promoting angiogenesis, and restoring electrical conduction [120]. Chitosan-based systems are also being engineered for in-stent restenosis and inflammation in atherosclerotic lesions. Catechol-modified chitosan films, when coassembled with hyaluronic acid and loaded with ACS14 (a hydrogen sulfide donor), respond to acidic microenvironments by releasing their therapeutic load to reduce smooth muscle proliferation, platelet adhesion, and inflammation, thereby offering a responsive alternative to conventional drug-eluting stents [121]. Also, chitosan has been employed in the development of cardiac patches for cardiomyopathy by incorporating selenium nanoparticles into its matrix, resulting in films that combine mechanical strength and conductivity while supporting cardiomyocyte proliferation and potentially modulating oxidative stress in damaged cardiac tissue [55].

In vascular tissue engineering, chitosan has been layered with heparin onto polyurethane coated decellularized scaffolds using a layer-by-layer approach, yielding polyelectrolyte multilayer patches that show hemocompatibility, reduced platelet adhesion, and improved endothelial progenitor cell proliferation. Long-term vessel openness observed in porcine models further confirms the promise of this approach for vascular graft applications [122]. In cardiac tissue engineering, chitosan blended with alginate and cardiac ECM resulted in natural ternary scaffolds with high porosity, improved mechanical integrity, and strong support for human mesenchymal stem-cell proliferation and cardiomyocyte differentiation, maintaining structural stability under physiological conditions [54]. Lastly, chitosan has been functionalized via catechol grafting and thiolene click chemistry employed in next-generation vascular stents, enabling the integration of zinc sulfate and REDV peptides. These coatings maintain nitric oxide release, promote endothelialization, inhibit thrombosis, reduce in-stent restenosis, and modulate inflammation, highlighting the versatility and therapeutic value of chitosan in cardiovascular interventions [123].

In a study conducted by Jiang et al. [124], galactose modified trimethyl chitosan nanoparticles (GTANPs) were developed for the targeted codelivery of atorvastatin, a statin and nucleic acid drug. These nanoparticles were engineered to achieve dual targeting in hepatocytes and lesional macrophages. The GTANPs demonstrated anti-inflammatory and lipid-regulating effects In vivo, and in ApoE-knockout atherosclerotic mice, they reduced plasma cholesterol, enhanced HDL-C levels, and decreased the atherosclerotic plaque burden. Especially, the combination of intravenous and oral administration routes enabled localized action in vascular lesions and systemic metabolic modulation.

In general, chitosan-based nanocarriers show a therapeutic potential for cardiovascular diseases; this is made possible by the mucoadhesive and biocompatible nature of chitosan, which can help to address pathological features such as inflammation, myocardial damage, fibrosis, and atherosclerosis through controlled release and tissue-specific action.

### 4.3. Applications in Neurodegenerative Disorders

**Figure 8:**
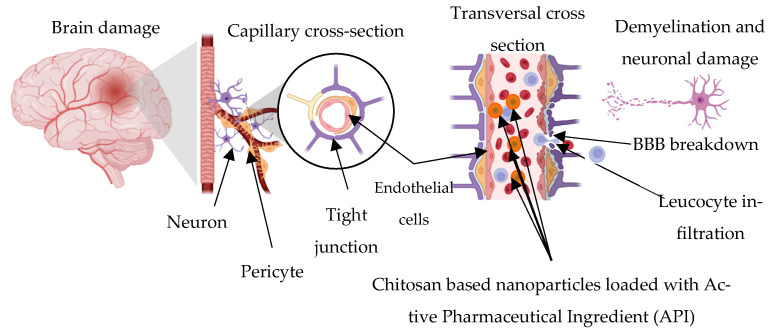
Chitosan-based nanoparticles used in applications in neurodegenerative disorders.

Ischemic stroke, which is a severe cerebrovascular event with neurodegenerative consequences, along with Alzheimer’s disease and Parkinson’s disease, presents significant therapeutic challenges, primarily due to the restrictive nature of the blood–brain barrier (BBB), which limits effective drug delivery to the central nervous system. Novelties in nanomaterial engineering, particularly those using chitosan nanoparticles, are overcoming these challenges by enabling the crossing of the BBB by temporarily opening tight junctions and leveraging their mucoadhesive properties and positive surface charge to enhance neuronal uptake of neuroprotective agents [57], as shown in Figure 8. This ability has helped to enable controlled and targeted delivery to the brain of a variety of therapeutics, including antioxidants, anti-inflammatory drugs, and gene therapies demonstrating neuroprotection and functional improvements in preclinical models [57,125,126].

Chitosan offers neuroregenerative applications, which have been tested in vitro by using amphiphilic chitosan oligosaccharide–cholesterol copolymers forming self-assembled micelles. These micelles have promoted neuronal differentiation by effectively delivering retinoic acid, a neurogenic agent, for neural tissue engineering [127]. Also, chitosan-stabilized liposomal nanoparticles encapsulating valproic acid have enhanced neuronal differentiation while minimizing teratogenic effects, supporting their application in neurodegenerative disease therapy and precision nanomedicine [128].

For the treatment of Alzheimer’s disease, chitosan-coated solid/lipid nanoparticles have been developed to encapsulate antioxidant drugs like ferulic acid, which is limited by its poor solubility, low BBB permeability, and extensive first-pass metabolism. Chitosan encapsulation for nose-to-brain delivery improves ferulic acid pharmacokinetics, providing mucoadhesive retention, sustained release, and effective transport directly to the brain. In preclinical Alzheimer models, this chitosan-coated solid/lipid nanoparticle achieved improved cognitive recovery, favorable biochemical changes, and better tolerability in animals, highlighting the potential for bypassing hepatic metabolism and delivering neuroprotective agents to the central nervous system [125]. Similarly, encapsulating sitagliptin, a DPP-4 inhibitor with central nervous system effects, in chitosan nanoparticles (SIT-CS-NPs) has demonstrated improved brain bioavailability, achieving more than 5-fold higher CNS drug levels upon intranasal administration compared to the free drug. These nanoparticles, around 188 nm in their average diameter and positively charged for enhanced mucosal adherence, also exhibited sustained release and reduced systemic exposure in targeted CNS drug delivery in Alzheimer’s disease [57].

In the case of Parkinson’s disease treatment, multifunctional chitosan–poly(ethylene glycol)–poly(lactic acid) (PEG-PLA) nanoparticles, conjugated with nerve growth factor (NGF), acteoside (ACT), and plasmid DNA (pDNA), have been shown in preclinical studies to cross the BBB, restore motor function, inhibit α-synuclein accumulation, and prevent dopaminergic neuron loss, key pathological features, [126,129]. In parallel, chitosan-coated niosomes loaded with anti-inflammatory drugs such as pentamidine inPentasomes have enabled non-invasive nose-to-brain drug delivery in PD models. These nanocarriers reduced neuroinflammation and rescued dopaminergic neuron populations in the substantia nigra and striatum by targeting glial activation and RAGE/NF-κB signaling, all while demonstrating stability and minimal systemic toxicity [129].

In the context of ischemic stroke, oxidative stress and BBB disruption are central to pathogenesis and block conventional drug delivery. Chitosan nanocarriers was engineered to exploit transient BBB permeability, offering targeted antioxidant therapy precisely to regions of excessive ROS production that develop during ischemia/reperfusion injury. These nanocarriers can be further surface-modified with ligands such as VCAM-1 or transferrin receptors to enhance selective binding to inflamed endothelium, thus increasing drug concentration at injury sites. Temporal BBB opening during early post-ischemic periods may be leveraged to improve paracellular transport, provided this is controlled to prevent secondary damage such as vasogenic edema. Codelivery strategies, for example, by combining ROS scavengers with agents like r-tPA or compounds that stabilize tight junctions, potentiate the neuroprotective effects [130].

In similar study, chitosan-coated nanoemulsions encapsulating rosmarinic acid (RA) were used to mitigate neuroinflammation and oxidative stress through nose-to-brain delivery, demonstrating neuroprotective effects against lipopolysaccharide-induced cognitive damage, neuroinflammation, and oxidative stress in Wistar rats. In intranasal administration of RA-chitosan-coated nanoemulsions, CNE enhanced RA bioavailability in the brain, unlike that of free RA, which, despite some biological effects, was not detectable in brain tissue. The RA CNE treatment improved memory function, suppressed neuroinflammatory markers, and promoted antioxidant protection by increasing cerebellar sulfhydryl content and reducing lipid peroxidation in the cortex [58].

Also, chitosan enhances olfactory neuronal differentiation by promoting maturation of olfactory receptor neurons, which may restore sensory function often weakened in the early stages of neurodegenerative diseases [131]. Similarly, the encapsulation of naringenin, a bioactive flavonoid with limited bioavailability, into chitosan nanoparticles (NAR-CNPs) reduced nose-to-brain delivery, improved cognitive function, decreased acetylcholinesterase activity, and reduced oxidative and neuronal damage in mouse dementia models [132].

These approaches have shown the functionality of chitosan nanoencapsulation for enhancing the delivery and efficacy of neuroprotective agents in neurodegenerative diseases in addressing the pathophysiology of cerebrovascular and neurodegenerative disorders, with the prospect of improving clinical outcomes via targeted, responsive, and synergistic therapies, enabling the effective transport of bioactive compounds via the nose-to-brain route.

### 4.4. Applications for Chronic Respiratory Diseases

**Figure 9:**
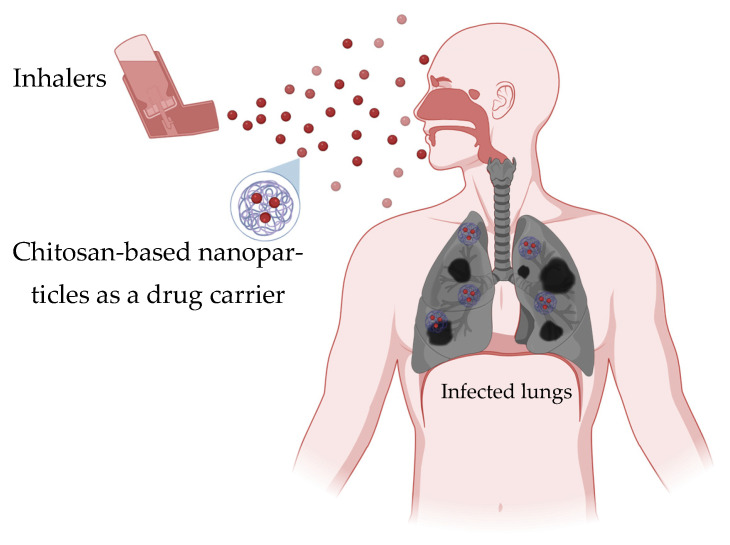
Chitosan-based nanoparticles used in applications in chronic respiratory diseases.

Asthma, chronic obstructive pulmonary disease (COPD), and cystic fibrosis are targets for inhalable chitosan-based nanomedicines in efforts to improve drug bioavailability and reduce inflammation. These conditions are characterized by permanent airway inflammation, mucus overproduction, and airflow limitation, requiring sustained and localized therapeutic interventions. Chitosan’s mucoadhesive nature enhances drug retention time in the respiratory tract, improving local bioavailability and reducing the need for frequent dosing. Its biodegradability and low toxicity make it suitable for pulmonary administration, especially in the form of nanoparticles, microspheres, or inhalable dry powders. Also, chitosan can encapsulate and ensure the controlled release of anti-inflammatory agents, bronchodilators, corticosteroids, and nucleic acids, aiding the modulation of immune responses and reducing oxidative stress in lung tissues. Functionalized chitosan carriers can also target specific cell types within the lungs, enhancing therapeutic precision and minimizing systemic side effects. Figure 9 demonstrates the use of chitosan-based nanoparticles in chronic respiratory diseases.

In a recent study, budesonide was incorporated into chitosan-based swellable microparticles designed for pulmonary administration, showing prolonged therapeutic effects in an allergic asthma animal model. These microparticles, tailored with different chitosan molecular weights, exhibited controlled in vivo release profiles that closely correlated with in vivo efficacy, maintaining therapeutic levels for up to 18 h after a single administration. Repeated dosing over seven days enhanced anti-inflammatory outcomes, as evidenced by reduced eosinophil counts and suppressed IL-4 and IL-5 expression in bronchoalveolar lavage and lung tissues. Significantly, the chitosan formulation enabled a reduction in dosing frequency to every two days and reduced the effective dose without compromising efficacy. These findings focus the potential of chitosan-based encapsulation to deliver respiratory therapeutics, reduce systemic exposure, and improve patient adherence in the management of chronic lung conditions such as asthma [59].

Similarly, ambrisentan was formulated into chitosan-coated LeciPlex nanoparticles (AMS-CTS-LPX), using a single-step process guided by a central composite design. The resulting nanoparticles had shown small particle size (~137 nm), a positive surface charge (+43.65 mV), and high entrapment efficiency (82.39%), which contributed to a sustained drug release of over 90% within 8 h. Importantly, in vivo pharmacokinetic studies in rats exposed a remarkable 5.6-fold increase in AMS bioavailability compared to conventional oral administration, highlighting the enhanced lung-targeted delivery achieved by chitosan coating. Also, histopathological evaluations confirmed the safety of the intratracheally administered formulation. These findings underscore the promise of chitosan-coated nanosystems as effective pulmonary delivery platforms to improve pulmonary arterial hypertension (PAH) treatment outcomes by providing controlled release and improved drug absorption [133].

Furthermore, a novel stimuli-responsive nanocarrier was designed by incorporating chitosan linked with a nitrobenzene group and functionalized with sialic acid (SA), allowing targeted anchoring to E-selectin overexpressed on pulmonary arterial endothelial cells (PAECs) under hypoxic conditions. This chitosan-based encapsulation system loaded with the clinically used drug ambrisentan enhances targeted delivery and facilitates intercellular transport to pulmonary artery smooth muscle cells (PASMCs), responding specifically to the reductive hypoxic microenvironment characteristic of PAH. Compared to free ambrisentan, the nanoencapsulated formulation with SA-PEG2000-NH_2_ prolongs blood circulation time, improves drug retention at the lesion site, and achieves greater therapeutic results by preventing vascular remodeling and reversing systolic dysfunction. This chitosan-anchored nanocraft represents a strategy for effective and sustained PAH therapy [134].

In another study done by Guarino et al., [135] aerosolized nanoparticles carrying human recombinant VEGF and SDF (VEGFNP/SDFNP) were administered to rats with monocrotaline-induced PAH, resulting in significant improvements in pulmonary arterial pressure, vascular resistance, and right ventricular hypertrophy. These chitosan-based or similar nanoparticle carriers enabled delivery of VEGF and SDF to the lungs, delaying vascular remodeling and reducing vessel occlusion. Even though the treatment did not fully restore the loss of distal pulmonary vessels, it improved endothelial nitric oxide synthase (eNOS) expression and decreased markers of smooth muscle proliferation, indicating repair and improvement of endothelial function.

Chitosan-based delivery platforms offer a multipurpose and effective approach to progress in the treatment outcomes of chronic respiratory disorders. Chitosan encapsulation has demonstrated promise in improving the therapeutic efficacy and bioavailability of drugs for the treatment of pulmonary arterial hypertension (PAH).

### 4.5. Applications in Rheumatoid Arthritis and Other Autoimmune Diseases

**Figure 10:**
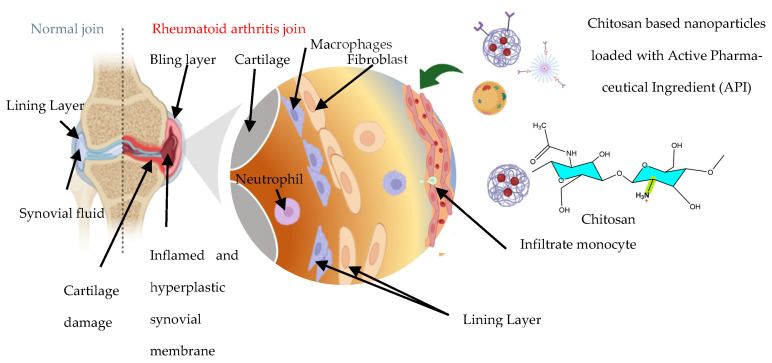
Chitosan-based nanoparticles used in applications in rheumatoid arthritis and other autoimmune diseases.

Chitosan-based encapsulation systems have also been developed as delivery platforms that enable the regulated release of various therapeutic agents for treating rheumatoid arthritis (RA) and other autoimmune conditions, as shown in Figure 10 and summarized in Table 4. These agents include anti-inflammatory drugs, immune system regulators, and chemical compounds such as methotrexate and indomethacin, as well as biological therapeutics like siRNA and monoclonal antibodies.

In a recent study, a multifunctional nanoplatform HA-M@PB@Ag@PD NPs integrating chitosan-silver with polydatin (PD), Prussian blue nanoparticles, hyaluronic acid, and a hybrid membrane was deployed for targeted delivery as a method in the treatment of rheumatoid arthritis. Chitosan played a key role in inducing apoptosis of fibroblast-like synoviocytes (RA-FLS) and synergizing with PD to regulate inflammatory macrophages. The nanocarrier system demonstrated significant in vivo efficacy by scavenging ROS, promoting macrophage repolarization (from M1 to M2 phenotype), and reducing synovial inflammation and joint degradation in a rat model of RA. The presence of chitosan not only enhanced the therapeutic activity but also helped reduce the required dosage, improving safety and pharmacokinetics. This targeted and sustained-release strategy highlights the potential of chitosan encapsulation in advancing RA therapy by directly modulating the synovial microenvironment and protecting joint integrity [71].

Similarly, chitosan-based encapsulation for oral delivery in the treatment of rheumatoid arthritis was developed in core–shell structures using a combination of self-micellization and ionotropic gelation techniques, incorporating chitosan as shell material. The resulting nanoparticles demonstrated enhanced encapsulation efficiency and sustained drug release at a pH of 6.8 for up to 48 h, while minimizing release at an acidic pH, thus protecting methotrexate (MTX) from premature degradation in the stomach. The pH-responsive behavior was attributed to the Pluronic F127–chitosan matrix, where chitosan formed the biocompatible outer shell, improving nanoparticle stability and targeting efficiency. In vivo studies in arthritic rats revealed improved bioavailability, extended circulation time, and enhanced accumulation of MTX at inflamed joints, compared to free MTX. Importantly, the chitosan-encapsulated MTX nanoparticles showed a positive safety profile with no observed toxicity, supporting their potential as a clinically relevant and effective approach for RA management [66].

Analogously, chitosan encapsulation of eugenol is a nano-herbal therapeutic strategy for the management of RA, offering an alternative to conventional treatments like methotrexate. In a study using a neonatal rat model of RA, eugenol encapsulated in chitosan nanoparticles demonstrated antioxidant, anti-inflammatory, and immunomodulatory effects. This nanocarriers reduced levels of malondialdehyde (MDA), a marker of oxidative stress, and downregulated the expression of pro-inflammatory genes such as TGF-β and MCP-1, comparable to the effects of methotrexate. Likewise, histopathological analysis confirmed reduced inflammation, synovial hyperplasia, and pannus formation in treated groups [63].

Chitosan encapsulation represents a valuable approach in the treatment of RA due to its natural carbohydrate-based properties and biocompatibility. As a biodegradable polymer, chitosan can form micro- and nanoparticles (M/NPs) that enhance the bioavailability, stability, and targeted delivery of anti-arthritic drugs. Given the complex RA microenvironment, characterized by overproduction of inflammatory cytokines, immune cell infiltration, and oxidative stress, chitosan-based M/NPs can be engineered to respond to stimuli such as pH and ROS, enabling on-demand and site-specific drug release. Therefore, the consistent demonstration of reduced inflammation, improved joint healing, decreased systemic side effects, and enhanced drug retention at target sites across multiple studies emphasizes the clinical promise of these systems. As research continues to advance, chitosan-based encapsulation strategies are poised to revolutionize RA treatment by offering safer, effective, and patient-friendly therapeutic options for managing this debilitating autoimmune disease [63].

In summary, these studies show the potential of chitosan-based nanocarriers in stimuli-responsive delivery of anti-arthritic therapeutics, enhancing bioavailability, joint targeting, and drug stability, demonstrating reduced inflammation, oxidative stress, and joint damage, while enabling tailored management of rheumatoid arthritis and other autoimmune diseases and minimizing systemic side effects.

### 4.6. Applications for Chronic Kidney Disease

**Figure 11:**
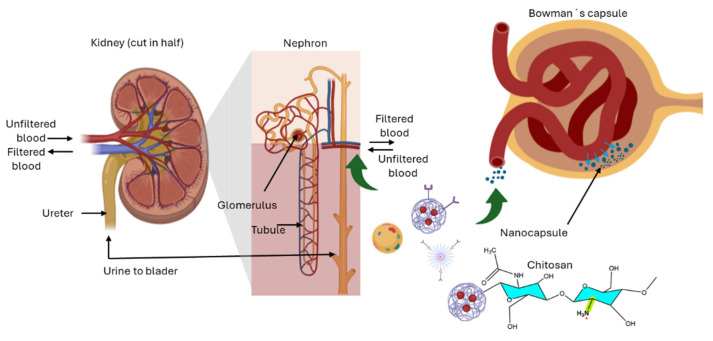
Chitosan-based nanoparticles used in applications in chronic kidney disease.

Nanoparticles can deliver therapeutic agents to the kidneys, reducing systemic toxicity. Chronic kidney disease (CKD) progression involves inflammation, fibrosis, and oxidative stress that led to gradual loss of renal function, requiring effective and targeted treatment strategies. Chitosan biocompatibility and biodegradability, combined with its ability to form nanoparticles and hydrogels, make it an ideal carrier for delivering anti-fibrotic agents, antioxidants, and anti-inflammatory drugs directly to renal tissues. These delivery systems can enhance drug stability, control release, and improve bioavailability, helping to maintain therapeutic drug concentrations over continued periods, as shown in Figure 11. Besides, chitosan mucoadhesive properties and the capacity for surface modification enable targeted delivery to specific kidney cells, potentially reducing systemic toxicity and side effects. By facilitating localized and sustained drug delivery, chitosan carriers offer a platform to slow CKD progression, mitigate renal damage, and improve patient outcomes.

Chitosan and its derivatives are a therapeutic tool for CKD, as found in the literature, offering renoprotective, anti-inflammatory, antioxidant, and targeted delivery capabilities. Carboxymethyl chitosan oligosaccharide (CMCOS), which has demonstrated significant renoprotective activity in animal models of adriamycin-induced nephropathy, mitigating inflammation, fibrosis, and oxidative stress while alleviating proteinuria, hypoalbuminemia, hyperlipidemia, renal hypertrophy, and histological damage. Mechanistic studies revealed that CMCOS reduces macrophage infiltration, suppresses myofibroblast transdifferentiation, prevents podocyte apoptosis, modulates proinflammatory (IL-1β, TNF-α) and profibrotic (TGF-β1) cytokines, and enhances antioxidant enzyme activities such as superoxide dismutase (SOD) and glutathione peroxidase, making it a biocompatible and non-toxic alternative to traditional hormone therapies [49].

Moreover, metformin-loaded chitosan nanoparticles prepared via ionic gelation exhibited excellent gastric stability, activated AMPK (AMP-activated protein kinase), reduced ENaC (epithelial sodium channel) currents, and lowered cyst burden in polycystic kidney disease models while maintaining normal renal function and improving systemic drug exposure, highlighting the potential for sustained-release, non-invasive therapies [76]. Similarly, mangiferin–chitosan nanoparticles enhanced the solubility, stability, and antioxidant efficacy of mangiferin, protecting kidney epithelial cells against sodium fluoride-induced oxidative damage by preserving intracellular antioxidant enzyme levels and preventing lipid and protein oxidation, positioning chitosan encapsulation as an antioxidant delivery platform [79]. It has also demonstrated renoprotective effects against cisplatin-induced nephrotoxicity, outperforming bone marrow-derived mesenchymal stem cells by reducing kidney dysfunction, oxidative stress, inflammation, fibrosis, and apoptosis while restoring antioxidant defenses, illustrating the multifunctional therapeutic potential of chitosan nanomedicines [78]. As for the nanocapsule form, it further enables targeted gene therapy in CKD, particularly for podocyte injury in idiopathic nephrotic syndrome, by delivering plasmids encoding microRNA against c-mip to reduce proteinuria, representing a pioneering approach for gene-regulated renal diseases [77].

Similarly, dual chitosan-coated liposomes encapsulating emodin, combined with an in situ colonic gel enema, improved oral bioavailability, cellular uptake, and gut microbiota modulation, resulting in attenuated renal fibrosis in unilateral ureteral obstruction models [82]. N-octyl-O-sulfate chitosan nanoparticles loaded with chrysophanol exhibited high encapsulation efficiency, sustained release, enhanced renal accumulation, and significant reductions in BUN (blood urea nitrogen), serum creatinine, and kidney injury molecule-1 (KIM-1), demonstrating robust nephroprotective and anti-fibrotic effects [81].

All in all, chitosan and its derivatives serve as both direct therapeutic agents and advanced delivery platforms in CKD, targeting key pathological mechanisms such as oxidative stress, inflammation, fibrosis, and podocyte injury, while improving drug stability, bioavailability, and patient compliance. These outcomes emphasize the broad potential of chitosan-based strategies for the prevention and management of CKD.

### 4.7. Applications for Infectious Diseases with Chronic Course

For diseases like HIV/AIDS, tuberculosis, hepatitis, and certain persistent bacterial or viral infections, chitosan-based nanomedicine drug delivery systems offer significant advantages through improved antiviral drug delivery and sustained release in the treatment of infectious diseases with a chronic course. These conditions require prolonged and consistent administration of antimicrobial or antiviral agents, which is often challenged by poor drug bioavailability, systemic toxicity, and patient non-compliance. Chitosan nanoparticles can be engineered to target infected cells or tissues, enhancing therapeutic efficacy while minimizing side effects. By overcoming physiological barriers and enabling sustained, targeted delivery, chitosan carriers contribute to effective management of chronic infectious diseases, potentially reducing drug resistance and improving patient adherence.

Chitosan has emerged as a polymer in the fight against infectious diseases, offering applications in drug and vaccine delivery. Infectious diseases such as tuberculosis and AIDS remain difficult to treat due to pathogen variability and frequent mutations, underscoring the need for new therapeutic strategies. Chitosan-based formulations address these challenges through their tunable properties, including size, charge, and functionalization, which enhance drug stability, bioavailability, and targeted delivery [14].

Nanoparticles derived from chitosan have demonstrated encouraging prospects for creating nanovaccines against infectious diseases, especially for HIV. Because peptide antigens alone exhibit low immunogenicity, innovative delivery systems are being explored to enhance the effectiveness of this approach. One such strategy involves the covalent attachment of HIV peptide antigens, specifically protease cleavage site peptide PCS5, to polysaccharide-based nanoparticles such as chitosan and hyaluronic acid, followed by association with oppositely charged polymers like dextran sulfate or chitosan, and the immunostimulatory molecule poly(I:C). These engineered nanosystems have demonstrated the ability to draw strong humoral immune responses, with PCS5-conjugated nanoparticles co-delivered with poly(I:C) inducing particularly robust activation of antigen-presenting cells. Remarkably, T cell activation kinetics varied depending on nanoparticle composition and mode of antigen attachment, suggesting that parameters such as polysaccharide type, antigen conjugation method (ionic, cleavable, or noncleavable), and adjuvant inclusion critically influence the balance between humoral and cellular immunity [138].

Chitosan-based polyelectrolyte complexes (PECs) have emerged as nanocarriers for infectious disease therapies, particularly in HIV treatments combining chitosan with chondroitin sulfate (ChonS) and stabilizing the system with zinc (II) ions. Researchers developed nanoPECs with excellent colloidal stability under physiological conditions. These nanoparticles were capable of encapsulating the antiretroviral drug tenofovir (TF) without compromising stability and could be further functionalized with antibodies such as anti-α4β7 to enhance targeting while maintaining biorecognition. Importantly, the formulations were noncytotoxic to human peripheral blood mononuclear cells (PBMCs) and demonstrated antiviral activity, reducing HIV-1 infection in a dose-dependent manner. The incorporation of zinc (II) contributed synergistically to infection inhibition. Compared to free TF, encapsulated drug in nanoPECs showed a lower IC50 and greater antiviral efficacy [139].

Chitosan-based nanomaterials have been researched as delivery carriers and adjuvants for DNA vaccines in the prevention of infectious diseases through mucosal immunization strategies. Because mucosal surfaces are the primary entry points for many pathogens, intranasal vaccination using modified chitosan derivatives such as *N*-2-hydroxypropyl trimethylammonium chloride chitosan (*N*-2-HACC) and *N*,*O*-carboxymethyl chitosan (CMC) offers a route to induce both systemic and mucosal immune responses. Nanoparticles prepared with these polymers demonstrated high encapsulation efficiency, stability, and safety, while effectively protecting plasmid DNA against degradation and enabling sustained release. Intranasal administration of *N*-2-HACC-CMC nanoparticles carrying a Newcastle disease virus (NDV) DNA vaccine improved IgG and sIgA antibody production, promoted lymphocyte proliferation, and increased cytokine levels (IL-2, IL-4, IFN-γ), reflecting balanced Th1 and Th2 immune activation. The mucoadhesive and permeation-enhancing properties of chitosan facilitated nanoparticle adhesion to the nasal epithelium and improved antigen uptake, resulting in stronger and longer-lasting immune responses, compared to intramuscular delivery [140].

Chitosan has shown promise in the development of targeted RNA interference (RNAi) therapies for infectious diseases through its role as a carrier for small interfering RNA (siRNA). While conventional chitosan nanoparticles enable gene delivery, their efficiency is limited by nonspecific uptake. To overcome this, researchers have modified chitosan with T cell-specific antibodies, such as CD7 single-chain antibodies (scFvCD7), to enhance targeted delivery. These antibody-conjugated chitosan nanoparticles form stable complexes with siRNA, maintaining favorable size and charge while improving cellular association with CD4+ T cells. Importantly, this targeted system demonstrated gene silencing efficiency, reducing CD4 receptor expression levels in T cells compared to nonmodified nanoparticles [141].

Furthermore, chitosan encapsulation of phytoconstituents such as gingerol addresses key therapeutic challenges in chronic respiratory infections, including poor bioavailability, limited aqueous solubility, and rapid elimination. The resulting chitosan–phytosome complex has shown stability, sustained release, and antioxidant, antibacterial, and anti-inflammatory activities, as well as oral absorption and prolonged therapeutic effects, in vivo [60]. In the same manner, chitosan and its derivatives combined with iron oxide nanoparticles (IONPs) have been developed as hybrid antiviral coatings for infectious disease control against coronavirus. These biopolymer–IONP coatings on cotton fabrics achieved a rapid virucidal activity of about 99% within 5 min for *N*-succinyl chitosan formulations and complete viral inactivation within 24 h, and demonstrated excellent stability, biocompatibility, and safety profiles compared to conventional toxic antimicrobial agents [142]. In another study, chitosan derivatives such as *N*-2-hydroxypropyl trimethyl ammonium chloride chitosan (HACC) have been developed as non-integrative gene delivery systems for antiviral applications. HACC/NbMLP28 nanocomplexes have assisted efficient plasmid protection, cellular uptake, and rapid resistance to multiple plant RNA viruses, offering a less damaging alternative to conventional methods [143]. Biotinylated chitosan nanoparticles similarly enabled targeted delivery of SARS-CoV nucleocapsid DNA to dendritic cells via DEC-205 receptor [144].

Overall, chitosan-based nanomedicine delivery systems represent a transformative innovation in managing chronic infectious diseases by addressing therapeutic challenges that have long hindered treatment efficacy. As research continues to refine these delivery platforms, chitosan-based nanomedicine holds promise for revolutionizing the treatment picture of chronic infectious diseases, eventually contributing to better patient outcomes, reduced healthcare burdens, and an effective approach to preventing drug adverse effects.

### 4.8. Applications in Diabetes Management

**Figure 12:**
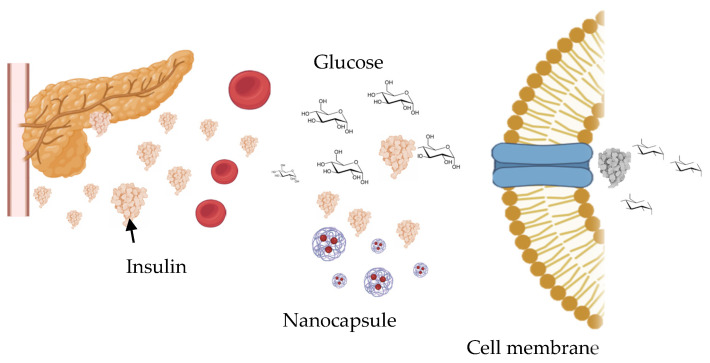
Chitosan-based nanoparticles used in applications in diabetes management.

Diabetes treatment remains challenging due to limitations in current therapies, poor oral bioavailability of peptide drugs, low patient compliance, and the multifactorial nature of diabetes-related complications. In response, chitosan-based encapsulation systems are able to address multiple aspects, ranging from oral insulin delivery and natural anti-diabetic compounds to wound healing strategies (Figure 12). A dominant and unifying theme among these studies is the mucoadhesive and absorption-enhancing properties of chitosan, which enable drug retention, epithelial interaction, and transepithelial transport. This is evident in oral insulin delivery systems, where chitosan-based nanoparticles protect insulin from gastrointestinal degradation while assisting intestinal absorption. Advanced strategies include bile acid–conjugated chitosan nanoparticles, which exploit apical sodium-dependent bile acid transporter (ASBT)-mediated endocytosis and intracellular trafficking via IBABP, achieving an oral insulin bioavailability of 15.9% and a pronounced hypoglycemic effect in diabetic rats [145]. Similarly, trimethyl chitosan/fucoidan nanoparticles combine strong mucoadhesion with paracellular transport enhancement and α-glucosidase inhibition, providing dual control of blood glucose through improved insulin bioavailability and delayed carbohydrate digestion [89].

Complementary approaches also highlight the versatility of chitosan in oral insulin delivery. Thiolated chitosan nanoparticles leverage mucus interaction and macropinocytosis-mediated uptake to prolong intestinal insulin residence and significantly reduce blood glucose levels in vivo [50]. Chitosan–mucin nanoparticles prepared via self-gelation demonstrate prolonged plasma insulin retention, low clearance, and good biocompatibility, outperforming free oral insulin solutions in diabetic models [85]. Additionally, chitosan-coated zein–carboxymethylated amylose nanocomposites exhibit up to a 12-fold increase in transepithelial permeability and relative bioavailability exceeding 15%, confirming the critical role of chitosan coatings in stabilizing insulin and promoting epithelial transport [146].

Beyond peptide therapeutics, chitosan encapsulation enhances the bioavailability and therapeutic efficacy of natural anti-diabetic compounds. Capsaicin-loaded chitosan nanoparticles embedded in cellulose acetate membranes show an encapsulation efficiency of 97% and effective α-amylase inhibition, supporting glucose regulation and antimicrobial and anticancer activity [147]. Similarly, quercetin-loaded alginate–succinylated chitosan core–shell nanoparticles improve glucose homeostasis and preserve renal and cardiac tissue integrity in diabetic rats, demonstrating protection compared to free quercetin [87]. Ferulic acid encapsulated in chitosan nanoparticles exhibits prolonged systemic retention and sustains antihyperglycemic effects, effectively mitigating oxidative stress and metabolic imbalance in diabetic models [84]. Chitosan-based systems combined with gold nanoparticles extend functionality by scavenging free radicals and inhibiting α-amylase and α-glucosidase, offering a multifunctional approach for metabolic control and infection management [90].

Another critical area addressed by chitosan encapsulation is diabetic wound healing, in which impaired tissue repair and chronic infection pose severe clinical challenges. Chitosan–fucoidan nanoparticles loaded with moxifloxacin and incorporated into pullulan-based microneedle patches provide sustained antibacterial delivery, rapid analgesia, and hemostasis, accelerating wound closure and reducing inflammation in diabetic mouse models [91]. Similarly, melatonin-loaded lecithin–chitosan nanoparticles enhance angiogenesis, fibroblast proliferation, and collagen deposition, leading to faster wound healing in diabetic rats [88]. Advanced hydrogel systems, such as synthetic chitosan-based composite hydrogels incorporating silver ions and EGF, further demonstrate the multifunctionality of chitosan by combining antimicrobial activity, growth factor delivery, and hydration capacity, achieving up to 97% wound closure and complete tissue regeneration in diabetic models [148].

One can conclude from the previous studies that chitosan functions as a multifunctional carrier material in diabetes management, providing mucoadhesion, protection of labile therapeutics, controlled and sustained release, enzyme inhibition, and tissue regenerative support. Its adaptability across nanoparticles, nanocomposites, membranes, microneedles, and hydrogels positions chitosan-based encapsulation systems as a powerful and integrative strategy for addressing both glycemic control and diabetes-associated complications.

### 4.9. Applications for Ocular Inflammation

**Figure 13:**
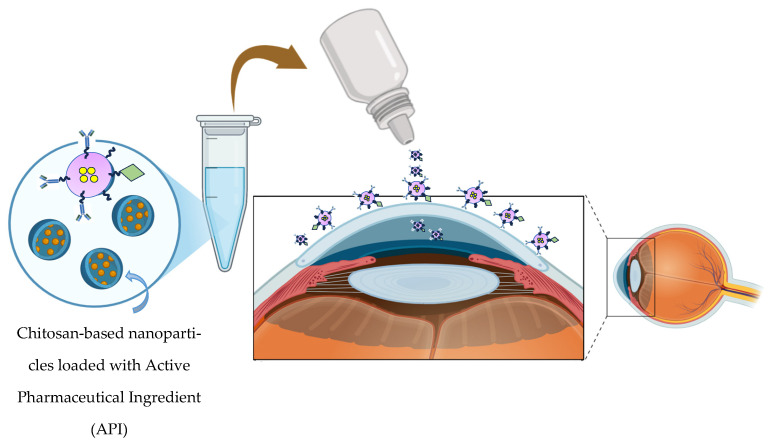
Chitosan-based nanoparticles used in applications in ocular inflammation.

Retinal diseases and diabetic retinopathy are leading causes of blindness, but treatment options are limited due to the biological barriers that delay drug delivery [17]. Polymeric materials are used in contact lenses, corneal and scleral implants, intraocular lenses, and vitreous substitutes, providing vision correction, tissue replacement, and the potential for wearable electronics or sensors. Polymers enable targeted delivery and sustained release of therapeutics, improving treatment of eye injuries, infections, and age-related degeneration. Advances in synthetics and biopolymers are enhancing functionality, biocompatibility, and versatility, though challenges remain in optimizing the performance, safety, and long-term effectiveness of retinal and intraocular therapies [149]. Figure 13 demonstrates the mechanism of chitosan-based nanoparticles in ocular inflammation treatment, and Table 5 shows relevant therapeutic outcomes from chitosan-based nanocarrier system tested in the In vivo/In vitro model.

A recent study developed resveratrol-loaded chitosan-coated niosomes (Chitoniosomes) as a novel nanoplatform for topical ocular delivery to treat inflammation. Resveratrol, a natural polyphenol, reduces proinflammatory cytokines TNFα and IL-6, which are key in eye inflammation that can cause irreversible tissue damage. Chitoniosomes were prepared using the ethanol-injection method with a surfactant blend of Span 60 and Poloxamer 407, coated with 0.6% chitosan. The optimized formulation (RSV.CsNiPolx) exhibited a particle size of 363.1 nm, positive zeta potential (+17.9 mV), high entrapment efficiency (90.3%), and sustained drug release over 24 h. Chitosan coating enhanced mucoadhesive efficiency by 1.9 times compared to uncoated niosomes, suggesting longer ocular residence and improved bioavailability. The formulation was stable for six months at 4 °C and showed ocular tolerance in rabbits without causing inflammation. In vivo studies demonstrated significant anti-inflammatory effects, with TNFα and IL-6 gene expression reduced by 49% and 55%, respectively, after three days of treatment [94].

Similarly, chitosan-coated poly (lactic-co-glycolic acid) (PLGA) nanoparticles (NPs) encapsulating the corticosteroid triamcinolone acetonide (TA) have been developed to enable sustained and controlled drug release via topical application. The chitosan coating enhances the mucoadhesive properties of the NPs, improving drug bioavailability by prolonging retention on the ocular surface. Optimized formulations of these NPs demonstrated consistent particle sizes of approximately 334–386 nm, positive surface charges (+26 to +33 mV), and encapsulation efficiencies between 55–57%. The chitosan coating also improved the thermal stability of the formulation and facilitated controlled release of TA over 27 h. These characteristics, coupled with the biodegradable and biocompatible nature of PLGA and chitosan, make this nanocarrier system a candidate for non-invasive ocular drug delivery [97].

Chitosan-based encapsulation systems have been studied across ocular pathologies, like corneal abrasion, dry eye disease, glaucoma-associated inflammation, and both anterior and posterior ocular inflammation. Despite differences in disease etiology and formulation design, these studies converge on several shared mechanisms by which chitosan enhances therapeutic outcomes [95,96,150,151]. The cationic and mucoadhesive nature of chitosan improves ocular surface retention by electrostatic interaction with the negatively charged mucin layer of the tear film and corneal epithelium. This property is evident in chitosan-based hydrogel sheets developed for corneal abrasion treatment [150], chitosan-coated nanoemulsions for dry eye disease [151], phytocubosomes for glaucoma-associated inflammation [96], and chitosan-modified nanostructured lipid carriers for genistein delivery [95]. Those chitosan-based systems solubilize and stabilize poorly water-soluble anti-inflammatory agents, such as ibuprofen [151], luteolin [96], and genistein [95]. Chitosan as a surface coating or matrix incorporation offers good drug entrapment, stability, and controlled or sustained release profiles, which are beneficial in ocular tissues, where rapid clearance limits drug efficacy. Chitosan encapsulation also demonstrates multifunctional therapeutic strategies, combining anti-inflammatory, antioxidant, wound-healing, and anti-scarring effects [95,96,150,151].

Across all formulations, biocompatibility and ocular safety were consistently demonstrated through in vivo and ex vivo models, reinforcing the suitability of chitosan for ophthalmic applications [95,96,150,151]. Whether incorporated into hydrogels, nanoemulsions, cubosomes, or thermosensitive in situ gels, chitosan-based carriers-maintained nanoscale size, favorable surface charge, and compatibility with sensitive ocular tissues. In general, these studies highlight chitosan’s role as a key functional excipient that enhances mucoadhesion, prolongs corneal residence, enables sustained and multistage drug release, and improves therapeutic efficacy in ocular inflammation. Its adaptability across multiple delivery platforms makes chitosan a unifying and powerful material useful for addressing both acute and chronic inflammatory conditions of the eye [95,96,150,151].

## 5. Challenges and Future Perspectives

Chitosan-based nanocarrier previous examples have shown promise in drug delivery and therapeutic applications. Significant challenges continue to delay chitosan’s application in the translation from laboratory research to general clinical use. These limitations limit technical, manufacturing, and regulatory domains, and each requires careful consideration and innovative solutions.

First, and importantly, natural source heterogeneity affects DD, MW, and impurity profiles, complicating reproducibility and regulatory approval. The scale-up of nanocarrier production from the laboratory to industrial phase often alters the essential characteristics such as particle size, colloidal stability, and drug release profiles. Achieving consistent encapsulation efficiency, batch reproducibility, and product shelf-life during large-scale manufacturing remains an obstacle. Stability issues are also common; several nanosystems are prone to aggregation, degradation, or premature load leakage during storage or circulation. Additionally, the ability to cross physiological barriers, such as the blood–brain barrier, remains limited for most nanoencapsulated systems, but as we saw here, it is possible to modulate the biomimetic encapsulation surface.

On the regulatory front, the lack of standardized guidelines for nanoformulated drugs, particularly those designed for personalized or stimuli-responsive delivery, adds ambiguity to the development pathway. Regulatory agencies like the FDA and EMA often assess each new nanosystem as a unique chemical entity, leading to increased analysis, time, and cost. Also, the clinical trial process for nanomedicines is long and expensive, with many candidates failing in late-stage trials due to suboptimal efficacy or unexpected toxicity. A key worry is the lack of long-term toxicity data for chronic exposure to nanomaterials, which increases regulatory caution and slows down approvals.

Economically, nanoencapsulation-based drug delivery systems are associated with high research and development costs, often exceeding those of traditional pharmaceuticals. This financial burden is intensified by the opinion that nanoformulations represent incremental, rather than innovative, innovations when they involve the reformulation of existing drugs. Consequently, many investors and pharmaceutical companies consider them low-return ventures, especially when their applications are limited to niche markets. As a result, funding is frequently directed toward conventional, lower-risk alternatives.

Finally, gaps in knowledge transfer and interdisciplinary collaboration delay progress. Effective nanoencapsulation requires coordinated efforts among chemists, materials scientists, biomedical engineers, pharmacologists, clinicians, and regulatory experts. The lack of integration delays innovation and slows translation from the lab to the industrial scale. Combined with this is the general lack of knowledge and training in nanomedicine among healthcare providers. Nano-drug delivery remains diminished in medical and pharmaceutical education, and few accessible resources exist to communicate its principles and benefits clearly to professionals or the public. Together, collaborative efforts between materials scientists, pharmaceutical researchers, regulatory experts, and clinical investigators will be essential to overcome these barriers and realize the full therapeutic potential of chitosan nanomedicines.

## 6. Conclusions

In conclusion, this review provides an overview of current advances in chitosan-based nanocarriers for drug delivery in chronic disease management. Beyond their role as passive carriers, chitosan-based systems enable tailored therapeutic strategies through tunable physicochemical properties, chemical functionalization, and intrinsic bioactivity, supporting personalized and disease-specific treatment approaches. Chitosan’s capacity for surface modification and stimulus responsiveness makes it attractive for targeted drug delivery across a range of pathological contexts.

However, effective system design requires selection and optimization of chitosan derivatives according to drug properties, administration route, and targeting strategy. Parameters such as degree of deacetylation, molecular weight, and type of chemical modification influence mucoadhesion, biodistribution, immunological interactions, and therapeutic performance. Aligning these with passive or active targeting approaches is essential to maximize efficacy while minimizing off-target effects and safety concerns.

Based on the integrated evidence, chitosan-based nanocarriers show promise in mucosal and localized delivery routes, including ocular, nasal, and pulmonary administration, as well as in immunomodulatory therapies requiring tunable immune interactions. In addition, stimulus-responsive systems exploiting pH or inflammatory microenvironments represent attractive strategies for altering structure–property relationships to improve site-specific drug release and therapeutic precision.

Advancing the clinical translation of chitosan-based nanomedicine will require harmonized material standards, improved control over batch-to-batch variability, and informed design frameworks. Future research should prioritize disease-specific optimization, standardized physicochemical characterization, and head-to-head comparisons with alternative delivery platforms to clearly define the contexts in which chitosan-based nanocarriers can offer clinically meaningful advantages.

## Figures and Tables

**Table 1:** Chitosan mucosal, immunomodulatory, and stimulus-responsive delivery compared to some other natural polymers.

Polymer	Surface Charge	Mucoadhesion	Immune Interaction	Degradability	Limitation	Ref.
Chitosan	Positive (+15 to +40 mV)	High	Tunable (stimulatory or suppressive)	Enzymatic (lysozyme)	Complement activation at high charge	[27,28,29,30,31,32,33,34,35,36,37,38]
Alginate	Negative	Moderate	Limited	Ionic exchange	Weak cell interaction	[39]
Dextran, Dextran sulfate	Neutral	Low	Inert	Enzymatic	Limited targeting capability	[40,41]
Hyaluronic acid (HA)	Negative	Moderate	MW-dependent	Enzymatic	Non-specific protein absorption	[42]

**Table 2:** Chitosan structure in the design of chitosan-based nanocarriers.

Chitosan Parameter	Benefit	Limitation	Ref.
High DD	↑ Mucoadhesion, ↑ Cellular uptake	↑ Complement activation, ↑ Coagulation risk	[98]
High MW	↑ Barrier retention	↓ Tissue penetration, Slower clearance	[99,100]
Chitosan chemical functionalization	Dependent on chemical modification		[101]

**Table 3:** Summary of the chitosan-based nanocarrier system with the encapsulated active ingredients employed for targeting cancer treatments.

Nanocarrier System	Load	Features	Stimulus	In Vitro Model	In Vivo Model	Therapeutic Outcomes	Ref.
FA-PMAN/CS nanoparticles	Model anticancer agents	Biomimetic terpolymer (phosphorylcholine, sulfonic acid, active ester), chitosan; folate functionalization; size 100–200 nm	Active targeting (folate receptor); immune evasion	Rat breast cancer cells	Murine tumor models	5× longer blood half-life vs TPP/CS; 5–6× higher cellular uptake; tumor-specific biodistribution	[53]
CS-based pH-sensitive polymer–drug conjugates	Various anticancer drugs	Polymer–drug conjugation; pH-cleavable interactions	pH-responsive (acidic TME, endosomes/lysosomes)	Cancer cell lines (general)	—	Selective drug activation in acidic environments; reduced systemic toxicity; efficacy depends on polymer–drug chemistry	[115]
DOX-CS-NPs (CS/TPP)	Doxorubicin	Ionic gelation with TPP; pH-responsive chitosan matrix	pH-responsive (tumor pH 6.6)	HeLa cells	—	Enhanced DOX internalization; increased apoptosis and cell cycle arrest; stronger cytotoxicity at acidic pH	[116]
DIO@CS nanoparticles	Diosgenin	Encapsulation in chitosan; molecular weight-dependent release	Passive targeting, controlled release	A431 skin cancer cells	DMBA-induced rat mammary cancer	Improved bioavailability; reduced oxidative stress; tumor growth suppression; tissue structure restoration	[117]
F/CS@TPGOx nanocarriers	TMB, Prussian blue NPs, glucose oxidase	Fucoidan–chitosan matrix; multifunctional payload	TME targeting (P-selectin, glucose-rich TME); ROS, starvation, photothermal	Cancer cell lines	Tumor-bearing mice	Rapid cell killing (<20% viability in 4 h); strong in vivo tumor suppression; low systemic toxicity	[48]
HA-CS nanoparticles	Doxorubicin + miR-34a	HA/chitosan polyelectrolyte complex; co-delivery platform	CD44-mediated targeting; gene–drug synergy	Triple-negative breast cancer cells	Tumor-bearing mice	Bcl-2 downregulation; Notch-1 inhibition; enhanced apoptosis; reduced migration and metastasis	[52]
CS/siRNA interpolyelectrolyte complexes	siRNA (EGFP, BCR/ABL-1)	Electrostatic complexation; nanoscale particles	Gene silencing (RNAi)	Murine macrophages; H1299 lung cancer; K562 leukemia	Transgenic mice (nasal delivery)	Up to 90% gene silencing; effective non-invasive pulmonary delivery	[102]
T7-polyGIONs (CD-CS coated)	miR-100 + antimiR-21 + TMZ	Gold–iron oxide core; β-CD/chitosan coating; PEG-T7 functionalization	Transferrin receptor targeting; BBB crossing; Theragnostic	Glioblastoma (GBM) cells	Orthotopic GBM mouse model	Brain accumulation; sensitization to TMZ; prolonged survival	[118]

**Table 4:** Summaries of the chitosan-based nanocarrier system with the encapsulated active ingredients for targeting rheumatoid arthritis (RA) treatment.

Chitosan-Based Nanocarrier System	Encapsulated Active Ingredients	Particle Size/Zeta Potential	Relevant Results	In Vitro/In Vivo Model	Ref.
PEGylated lecithin+A2:D9-chitosan nanoparticles (LEF-PEG-LCNPs)	Leflunomide (LEF)	~115 nm/positive ζ	Loading about 90%; controlled release; reduced TNF-α, IL-6, paw edema; improved oral bioavailability and reduced hepatotoxicity	In vivo: RA rat model	[61]
Cholesterol-grafted chitosan nanoparticles conjugated with anti-folate receptor-β antibody (Ab-CCM-NP)	Methotrexate (MTX)	~181 nm/NR	Active macrophage targeting; prolonged MTX half-life; reduced paw swelling and pro-inflammatory cytokines; enhanced joint accumulation	In vivo: arthritic animal model (macrophage-rich synovium)	[64]
Folate-targeted chitosan–chondroitin sulfate nanoparticles incorporated into hydrogel with almond oil	Leflunomide (LEF)	NR/NR	FR-β–mediated macrophage targeting; pH-responsive release (max at pH 5); reduced cytokines, joint swelling, and pain with minimal toxicity	In vivo: RA animal model (transdermal delivery)	[65]
Chitosan-oxidized chondroitin sulfate-sodium glycerol β-phosphate thermosensitive hydrogel with apoferritin nanoparticles	siHMGB1	Hydrogel system/NR	Sustained intra-articular siRNA release; M1, M2 macrophage repolarization via HMGB1/TLR4/NF-κB inhibition; reduced synovial inflammation and joint damage	In vivo: RA animal model (intra-articular injection)	[67]
Hyaluronic acid–chitosan nanoparticles incorporated into composite microsphere system (NiMPs) with PLGA and PCADK polymers	siRNA targeting Myeloid cell leukemia-1 (Mcl-1)	NR/NR	Sustained siRNA release; protection from degradation; enhanced macrophage uptake; superior therapeutic efficacy with reduced dosing frequency	In vivo: adjuvant-induced arthritis rat model	[68]
Chitosan–magnetite nanoconjugates (MC-MNCs)	Meloxicam	~258 nm/NR	Magnetic targeting; sustained release; enhanced anti-inflammatory and anti-arthritic efficacy with reduced systemic toxicity	In vivo: murine arthritis and inflammation models	[69]
Chitosan transfersomal gel (DXI-TG)	Dexibuprofen (DXI)	Nanoscale transfersomes/NR	Enhanced skin permeation; ~5-fold bioavailability increase; sustained release; significant anti-arthritic efficacy without organ toxicity	In vivo: acute arthritis animal model (topical)	[70]
Chitosan biodegradable nanoparticles (DGN-NPs)	Diosgenin	~290 nm/positive ζ	Sustained release; superior anti-inflammatory and analgesic effects vs free drug and MTX; modulation of NF-κB, IL-6, TNF-α	In vivo: RA rat model	[72]
Chitosan-coated metal-organic framework nanoparticles (MIL-100(Fe))	Methotrexate (MTX)	NR/NR	Improved stability and macrophage uptake; controlled release; enhanced cytotoxicity toward activated macrophages; reduced systemic toxicity	In vitro: activated macrophages; In vivo: RA animal model	[73]
Tin oxide–Chitosan–Polyethylene glycol–Carvacrol nanocomposites (SCP-CAR)	Carvacrol	NR/NR	Reduced arthritis severity, cytokines (IL-6, IL-1β, TNF-α, COX-2); increased IL-10; improved histopathology and antioxidant status	In vivo: CFA-induced RA rat model	[74]
Chitosan-coated nanoliposomes	Indomethacin	NR/positive ζ	Loading about 99% mucoadhesion and stability; oral protection and controlled NSAID release	In vitro: formulation & stability studies; In vivo: NR	[75]
Folic acid-conjugated chitosan-coated metal-organic framework nanoparticles (FA-CS/UIO-66-NH2)	CO-releasing molecule (CORM)	NR/NR	Targeted delivery to folate receptor–positive cells; light-triggered sustained CO release; improved stability, imaging, and therapeutic control	In vitro: folate receptor–positive cells	[136]
Chitosan/β-glycerophosphate thermosensitive hydrogels with NLCs and PLGA nanoparticles (CS/βGP)	Leflunomide (LEF) and Dexamethasone (Dex)	Hydrogel depot/NR	Sustained intra-articular release (Dex 58 d; LEF 17 d); better joint retention; reduced TNF-α; significant histological recovery	In vivo: RA rat model (intra-articular)	[137]

NR: not reported in the original study.

**Table 5:** Summary of the chitosan-based nanocarrier system with the encapsulated active ingredients for targeting ocular inflammation treatments.

Chitosan-Based Nanocarrier System	Encapsulated Active Ingredients	Characteristics	Therapeutic Outcomes	In Vitro/In Vivo Model	Ref.
Chitosan-functionalized PLGA nanoparticles	Sirolimus	~300–400 nm; positive ζ due to chitosan; sustained release	Enhanced episcleral retention and retinal delivery after subconjunctival injection; reduced retinal inflammation, apoptosis, and photoreceptor loss without systemic toxicity	In vivo: retinal degeneration models (rodents; subconjunctival injection)	[80]
N-palmitoyl-N-monomethyl-N,N-dimethyl-N,N,N-trimethyl-6-O-glycolchitosan (MET) nanoparticles	Rapamycin	Cationic chitosan derivative; high corneal adhesion; topical eye-drop formulation	Effective delivery to choroid–retina; inflammation reduction comparable to dexamethasone; Th17 suppression and Treg activation in EAU model	In vivo: experimental autoimmune uveitis (EAU) mouse model	[92]
Chitosan nanoparticles incorporated in Poloxamer 407–gellan gum in situ hydrogel	Dexamethasone and antibiotics	~286 nm; ζ ≈ +20 mV; thermogelling, porous hydrogel matrix	Prolonged ocular retention; sustained release via diffusion/erosion; high cell viability; suitable for combined anti-inflammatory/antimicrobial therapy	In vitro: cytotoxicity assays; Ex vivo: ocular compatibility	[93]
Chitosan-coated nanostructured lipid carriers in thermosensitive in situ gel	Genistein	Nanometric size; ζ ≈ +25 mV; EE ≥ 98.8%; release up to 48 h (NPs)/120 h (gel)	Improved corneal and retinal IL-6 suppression, retention, controlled release and ocular biocompatibility	In vivo: ocular inflammation models (rodents)	[95]
Chitosan-coated phytocubosomes	Luteolin	Nanometric size; ζ positive; EE up to 96%; sustained 24 h release	3.6-fold increase in corneal permeation and IOP reduction and anti-inflammatory activity in glaucomatous rabbits	In vivo: glaucomatous rabbit model	[96]
Functional hydrogel sheet (THS) with chitosan, poly(hydroxyethyl methacrylate), and zinc oxide nanoparticles	Epigallocatechin gallate (EGCG), β-1,3-glucan	Solid mucoadhesive hydrogel; multistage release platform	Sequential drug release; >90% corneal healing; 8-fold recovery vs conventional therapy; reduced inflammation and scarring	In vivo: corneal abrasion animal model	[150]
Chitosan-coated nanoemulsions (NEs) with lecithin	Ibuprofen	Stable NEs; chitosan-induced positive surface charge; tear-film compatible	Prolonged ocular residence; improved NSAID delivery; potential dual role as drug carrier and artificial tear	In vitro/Ex vivo: ocular surface models	[151]
Chitosan-triphenylphosphonium (TPP) nanoparticles with phycocyanin and β-glucan	Lutein	Mitochondria-targeting TPP; mucoadhesive chitosan shell	2.36-fold increase in mitochondrial lutein; reduced ROS, inflammation, and keratinization in DED	In vivo: dry eye disease (DED) animal model	[152]
Chitosan–N-acetylcysteine (CS-NAC) conjugate-grafted nanostructured lipid carriers	Dexamethasone	Thiolated chitosan; enhanced mucin binding; stable NLC core	Improved tear film stability, corneal penetration, and anti-inflammatory efficacy vs Dex eye drops	In vivo: dry eye disease model; In vitro: ocular safety assays	[153]
Carboxymethyl chitosan-stabilized nanosuspensions in thermo-responsive in situ hydrogel	Fluticasone propionate (FP)	~275 nm; ~38 mV; sol–gel transition at 37 °C	Biocompatibility confirmed by Confirmed by cytotoxicity and eye-irritation tests	In vivo: blepharitis rat model	[154]
Chitosan nanoparticles	Anti-inflammatory and antibiotic agents	Tunable size and charge; mucoadhesive polysaccharide matrix	Prolonged ocular retention; reduced drug washout; potential for multi-drug ocular therapy	NR (general platform description)	[155]

NR: not reported in the original study.

## Data Availability

The data presented in this study is available on request from the corresponding author.
